# Effects of the two-pore potassium channel subunit Task5 on neuronal function and signal processing in the auditory brainstem

**DOI:** 10.3389/fncel.2024.1463816

**Published:** 2024-11-01

**Authors:** Mahshid Helia Saber, Michaela Kaiser, Lukas Rüttiger, Christoph Körber

**Affiliations:** ^1^Department of Functional Neuroanatomy, Institute of Anatomy and Cell Biology, Heidelberg University, Heidelberg, Germany; ^2^Molecular Physiology of Hearing, Tübingen Hearing Research Centre, Department of Otolaryngology, University of Tübingen, Tübingen, Germany

**Keywords:** cochlear nucleus, MNTB, ABR, auditory system, bushy cells, stellate cells

## Abstract

Processing of auditory signals critically depends on the neuron’s ability to fire brief, precisely timed action potentials (APs) at high frequencies and high fidelity for prolonged times. This requires the expression of specialized sets of ion channels to quickly repolarize neurons, prevent aberrant AP firing and tightly regulate neuronal excitability. Although critically important, the regulation of neuronal excitability has received little attention in the auditory system. Neuronal excitability is determined to a large extent by the resting membrane potential (RMP), which in turn depends on the kind and number of ion channels open at rest; mostly potassium channels. A large part of this resting potassium conductance is carried by two-pore potassium channels (K2P channels). Among the K2P channels, the subunit Task5 is expressed almost exclusively in the auditory brainstem, suggesting a specialized role in auditory processing. However, since it failed to form functional ion channels in heterologous expression systems, it was classified “non-functional” for a long time and its role in the auditory system remained elusive. Here, we generated Task5 knock-out (KO) mice. The loss of Task5 resulted in changes in neuronal excitability in bushy cells of the ventral cochlear nucleus (VCN) and principal neurons of the medial nucleus of the trapezoid body (MNTB). Moreover, auditory brainstem responses (ABRs) to loud sounds were altered in Tasko5-KO mice. Thus, our study provides evidence that Task5 is indeed a functional K2P subunit and contributes to sound processing in the auditory brainstem.

## Introduction

Mammalian auditory processing, especially sound source localization, relies on the ability of the lower auditory brainstem circuit to transmit information at high fidelity with sub-millisecond precision over a wide range of frequencies, up to several 100 Hertz (Grothe et al., 2010; Joris and van der Heijden, 2019). Auditory neurons are therefore able to fire brief (<1 ms), action potentials (APs) at high frequency for prolonged times (e.g., Kopp-Scheinpflug et al., 2008; Sonntag et al., 2009; Lorteije et al., 2009). This allows the maintenance of timing information with a typical spike timing precision of less than 1 ms. Neurons transmitting timing information often show an onset firing pattern when stimulated *in vitro* (e.g., Manis and Marx, 1991; Brew and Forsythe, 1995). Both, high frequency firing and onset firing pattern have been attributed to the expression of specific sets of ion channel subunits, which provide rapid AP repolarization (K_v_3 channels) and prevent the firing of multiple APs upon depolarization (K_v_1 and HCN channels) (Rothman and Manis, 2003; Oertel et al., 2008). However, high frequency firing also depends on the precise regulation of neuronal excitability and thus the RMP, which is set by all ion channels open at rest (Hodgkin and Huxley, 1952). Among these open channels, two-pore potassium channels (K2P channels) contribute the vast majority of the potassium leak conductance (Goldstein et al., 2001, 2005; Honoré, 2007).

K2P channels comprise a family of 15 subunits that are widely expressed in the central and peripheral nervous system (Goldstein et al., 2001). Since K2Ps conduct ions in a voltage-independent manner, they constitute potassium leak channels open at rest and thus contribute to the RMP (Honoré, 2007). Additionally, recent studies in HEK293 cells and trigeminal afferent nerves have shown that K2Ps participate in AP repolarization or might even mediate it entirely (MacKenzie et al., 2015; Kanda et al., 2019). Among the K2Ps, Task5 is almost exclusively expressed in the auditory brainstem (Karschin et al., 2001), suggesting a role in auditory signaling. However, the function of Task5 has long remained enigmatic since it failed to form functional homomeric or heteromeric (with Task1 or Task3) ion channels, in various heterologous expression systems (Ashmole et al., 2001; Kim and Gnatenco, 2001). Of note, a recent study suggested that Task5 forms heteromeric ion channel complexes with Task1 and Task3 which show reduced plasma membrane expression and altered gating as compared to the corresponding homomeric channels (Rinné et al., 2024). Moreover, Task5 expression correlates with processing of airborne sounds as it is strongly upregulated around the onset of hearing (Ehmann et al., 2013; Körber et al., 2014) and downregulated after hearing loss (Holt et al., 2006; Cui et al., 2007; Dong et al., 2009). Hence, the specific expression pattern of Task5 and its correlation with hearing ability suggest a role for Task5 in the processing of auditory information.

We investigated the role of Task5 in the regulation of the RMP and of AP firing in two nuclei of the auditory brainstem, the ventral cochlear nucleus (VCN) and the medial nucleus of the trapezoid body (MNTB), in early hearing mice. Neurons in both nuclei, the bushy cells of the VCN and the principal cells of the MNTB, contribute to sound source localization (Grothe et al., 2010) and show a phasic firing pattern (Rothman and Manis, 2003; Smith et al., 1998, respectively). Using a constitutive knock-out (KO) of Task5, we show that the absence of Task5 results in alterations in rheobase current in both types of neurons, which were accompanied by changes in input resistance and may thus indicate a functional compensation for the loss of Task5. However, these compensatory changes on the cellular level were incomplete, since we observed altered auditory brainstem responses (ABRs) to different tone stimuli, particularly at high sound pressure levels (SPLs), on the systemic level. Our results thus demonstrate a functional role of Task5 in auditory brainstem neurons involved in the processing of precisely timed auditory information.

## Materials and methods

### Animal and ethical approval

All experiments were conducted in accordance with the German federal law on the care and use of laboratory animals and the EU Directive 2010/63/EU for animal experiments. Protocols were approved by the local authorities (Regierungspräsidium Karlsruhe, Regierungspräsidium Tübingen). Constitutive, homozygous Task5 knock-out (KO) mice lacking the entire coding sequence of Kcnk15, the gene encoding Task5, were established from cryopreserved sperm [purchased from KOMP Repository (UC Davis, Davis, CA)] in the C57BL/6 N background. Homozygous Task5 KO mice were classified as “non-burdened.” KO animals used in the experiments were homozygous Task5 KO mice of either sex.

### Fluorescent *in situ* hybridization

Fluorescent *in situ* hybridization (FISH) against Task5 mRNA was performed at P7, P14 and P42 in WT and KO mice. Mice of either genotype were rapidly decapitated and brains were removed, snap frozen on aluminium foil on dry ice and stored at −80°C until further usage. 20 μm thick sections were prepared on a cryostat (Microm HM 560, Thermo Fisher). Sections were thaw-mounted on Super Frost Plus slides (Thermo Fisher), dried in the cryostat at −20°C and stored at −80°*C.* Prior to tissue preparation, the work area and all tools were cleaned with RNaseZAP (Sigma) to eliminate RNase contaminations.

FISH analysis was performed using the RNAscope Multiplex Fluorescent Reagent Kit (Advanced Cell Diagnostics, Newark, CA; Probe: Mm-Kcnk15-C2) according to the manufacturer’s instructions (freshly frozen tissue protocol, protease IV treatment: 15 min). FISH stained sections were briefly incubated in DAPI, mounted in Slow Fade Gold (Thermo Fisher) and stored at 4°C until imaging.

The presence of Kcnk15 (Task5) mRNA labelled by FISH was visualized by confocal microscopy. Confocal images were acquired on a Leica SP8 microscope equipped with a 63x HC PL APO (1.4 NA) objective (Leica).

### Electrophysiology

Mice were rapidly decapitated at P12-14. The brains were removed in ice-cold slicing solution containing (in mM): 125 NaCl, 25 NaHCO_3_, 2.5 KCl, 1.25 NaH_2_PO_4_, 3 myo-inositol, 2 Na-pyruvate, 0.4 ascorbic acid, 0.1 CaCl_2_, 3 MgCl_2_ and 25 glucose aerated with carbogen. Coronal slices containing the VCN and/or the MNTB were prepared on a vibratome (VT1200S, Leica) at a thickness of 200–300 μm. Slices were stored in ASCF containing (in mM): 125 NaCl, 25 NaHCO_3_, 2.5 KCl, 1.25 NaH_2_PO_4_, 2 CaCl_2_, 1 MgCl_2_ and 25 glucose aerated with carbogen (pH 7.3) at 37°C for 45 min and at room temperature (22 ± 1°C) thereafter.

Whole-cell patch-clamp recordings were established from neurons in the VCN and MNTB using an EPC-10/2 amplifier controlled by PatchMaster software (HEKA, Lambrecht, Germany) as described previously (Körber et al., 2014, 2015). Pipettes were pulled from borosilicate glass (Cat. no.: 1B150F-4, WPI, Sarasota, FL) and had open tip resistances of 3–8 MΩ. All recordings were performed at room temperature in ACSF. Recorded voltage traces were digitized at 100 kHz and Bessel-filtered (2.9 kHz). The pipette solution contained (in mM): 125 K-gluconate, 20 KCl, 5 Na_2_-phosphocreatine, 10 HEPES, 0.1 EGTA and 4 Mg-ATP and 0.3 GTP (pH 7.2). Recordings were performed at resting membrane potential and reported voltages have not been corrected for a liquid junction potential of-11 mV. Properties of APs and responses to prolonged depolarization were determined at 50 pA and 60 pA above threshold, respectively. Leak currents were recorded from a holding potential of-70 mV in ACSF supplemented with 1 μM tetrodotoxin (TTX, Hello Bio, Dunshaughlin, Ireland), 10 mM tetraethylamonium (TEA-Cl), 5 mM 4-aminopyridine (4-AP), 250 μM Cd^2+^ and 10 μM ZD7288 (Hello Bio) to block voltage-dependent sodium, potassium and calcium channels and HCN channels, respectively, digitized at 50 kHz and Bessel-filtered (2.9 kHz).

### Auditory brainstem responses

Auditory brainstem responses were recorded from both ears of Task5 KO mice and WT littermates at 6–8 weeks of age. Therefore, mice were anaesthetized [0.05 mg/kg Fentanyl (ratiopharm GmbH, Ulm, Germany), 0.5 mg/kg Medetomidine hydrochloride (Eurovet Animal Health B.V., Bladel, The Netherlands), 5 mg/kg Midazolam (Hameln Pharma plus GmbH, Hameln, Germany) and 0.2 mg/kg atropine (B. Braun, Melsungen, Germany)] and transferred to a soundproof chamber (IAC, Niederkrüchten, Germany). ABRs were measured as described previously (Rüttiger et al., 2013). In brief, electrical brainstem responses in response to click (100 μs), noise burst (1 ms) and pure tone stimuli (3 ms, including 1 ms cosine squared rise and fall envelope, 2–45.2 kHz) were measured using subdermal silver wire electrodes placed at the ear, vertex and back of the mouse. Recordings lasted for 10 ms with stimuli presented at alternating polarity to eliminate electrical stimulus artefacts. At each sound pressure level presented (usually 0–100 dB SPL in steps of 5 dB), signals were amplified (100,000-fold, 100 dB), bandpass filtered (0.2–5 kHz 6-pole Butterworth filter, Wulf Elektronik, Frankfurt, Germany) and averaged across 64–512 repetitions, depending on the signal to noise ratio. Recordings were digitized at 20 kHz. Sound stimuli were delivered to the ear in by a calibrated loudspeaker (DT-911, Beyerdynamic, Heilbronn, Germany) placed ~3 cm lateral to the animal’s pinna. Sound pressure was calibrated prior to each measurement using a microphone (B&K 4191, Bruel & Kjaer, Naerum, Denmark or MK301, Microtech, Gefell, Germany) placed near the animal’s ear.

Auditory thresholds were determined as the lowest SPL at which acoustically evoked potentials could be visually identified by an experimentally-blinded observer. The fine structure of the ABRs was analysed for waves, which were defined as consecutive amplitude deflections starting with negative peak and followed by a positive peak. Peak amplitudes of waves I–IV were extracted as described previously (Rüttiger et al., 2013) and input–output (I/O) growth functions were constructed for increasing stimulus levels relative to ABR thresholds.

### Statistical analysis

Data were analysed using custom written IGOR routines (Wavemetrics, Lake Oswego, OR), customized ABR analysis programs (Rüttiger et al., 2013) and Prism 10 (GraphPad, La Jolla, CA). Recordings obtained from Task5 KO mice were compared to those from WT mice as control. Statistical significance was determined using unpaired *t*-test (two groups, one parameter) or two-way ANOVA followed by Sidak’s multiple comparisons test (two groups, multiple parameters). The statistical test used is indicated in the respective section of the text. Data from patch-clamp recordings are displayed as mean ± sem, while ABR data are shown as mean ± sd. Significant differences are depicted using asterisks (*p* < 0.05 = *; *p* < 0.01 = **; *p* < 0.001 = ***).

## Results

Task5 has previously failed to produce measurable currents in various heterologous expression systems (Karschin et al., 2001; Ashmole et al., 2001). However, the function of Task5 has never been examined in its physiological context. To fill this gap, we established a constitutive knock-out of Task5 and investigated the effects of Task5 deficiency in neurons of the VCN and the MNTB, which express Task5 endogenously (Karschin et al., 2001; Ehmann et al., 2013; Körber et al., 2014).

### Generation of Task5 KO mice

**Figure 1:**
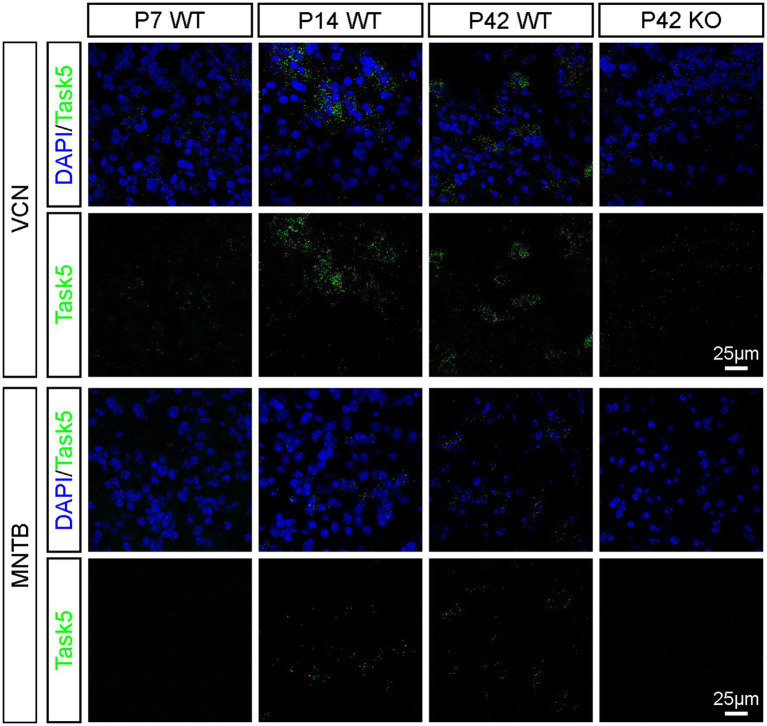
Expression of Task5 in the VCN and the MNTB is upregulated around the onset of hearing and stays high in adult mice. Task5 expression is not detected in KO mice (P42, right panels).

Homozygous Task5 KO mice (see Materials and Methods) were viable and fertile and showed no obvious phenotype. KO of Task5 was confirmed by fluorescent *in situ* hybridization (FISH). Example images taken from the VCN and MNTB of WT and KO mice are shown in Figure 1. In the VCN, Task5 mRNA was detected at all ages examined (P7, P14, P42), whereas KO littermates failed to show FISH signal for Task5 mRNA regardless of age (Figure 1). Prehearing (P7) WT mice showed only very weak Task5 mRNA expression in the VCN (Figure 1, upper row, left). However, Task5 expression increased substantially during neuronal maturation and resulted in strong FISH signals shortly after the onset of hearing (P14). There was no obvious change in the FISH signal for Task5 mRNA between P14 and P42, indicating that the expression level of Task5 was rather stable after the onset of hearing (Figure 1, P14 and P42). In the MNTB, Task5 mRNA labelling was readily detectable only at P14 and P42. We could not detect FISH signals for Task5 in the MNTB at P7 and in samples from KO mice. The expression pattern observed in the MNTB thus resembled the one in the VCN, albeit at lower levels (Figure 1). Moreover, the increase in Task5 expression around the onset of hearing in both nuclei is in agreement with previously published results from the VCN and the superior olivary complex (SOC) (Körber et al., 2014; Ehmann et al., 2013, respectively).

### Effects of Task5 on cellular properties of VCN neurons

To examine the effects of Task5 KO on the cellular properties of neurons in the VCN, we established recordings from large neurons with round-appearing somata located in the anterior VCN close to the auditory nerve root. Although the majority of neurons in this area are bushy cells (Lauer et al., 2013), which are recognized by their phasic firing pattern (one up to a few action potentials (APs) at the beginning of the stimulus), we also recorded a number of tonically firing neurons, most likely stellate cells (Wu and Oertel, 1984; Rothman and Manis, 2003). We classified the recorded cells as phasic or tonic firing cells based on their response to a prolonged current injection and analysed them separately (see below).

**Figure 2:**
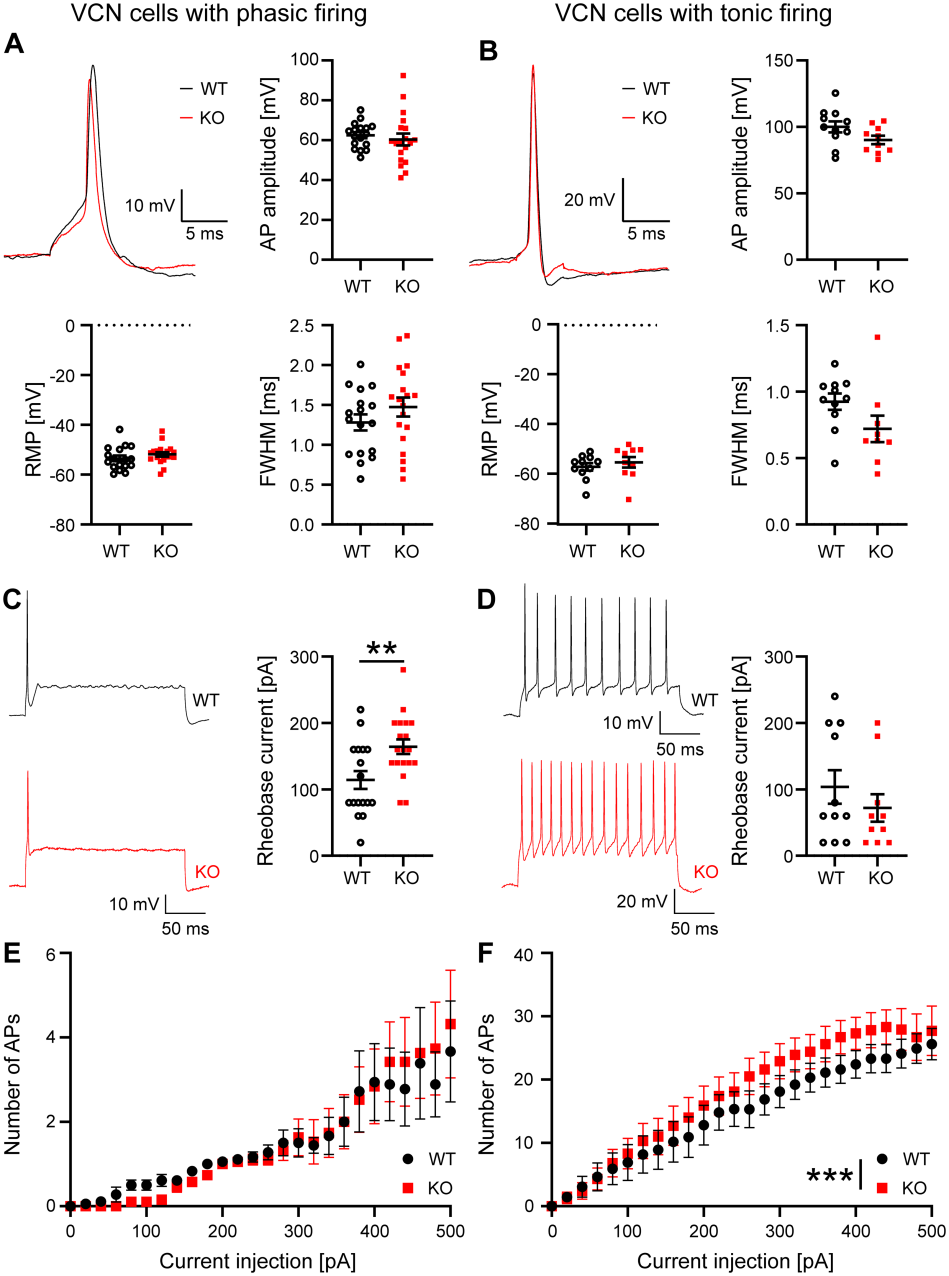
Cellular properties of phasically and tonically firing neurons in the VCN are mildly affected by Task5 KO. **(A,B)** Representative AP recordings from phasically **(A)** and tonically **(B)** firing VCN neurons and quantification of AP amplitude, resting membrane potential (RMP) and AP width (full width at half maximum (FWHM)). **(C,D)** Representative recordings of the neuronal firing pattern in response to prolonged depolarization (left) and quantification of the rheobase current (right). **(E,F)** Input–Output curves recorded from phasically and tonically firing VCN neurons. Data is presented as mean ± sem. ** = *p* < 0.01, *** = *p* < 0.001, Statistical testing: unpaired *t*-test **(A–D)** or two-way ANOVA followed by Sidak’s test for multiple comparisons **(E,F)**.

First, we investigated the waveform of APs elicited by short depolarizations (5 ms) in WT and Task5 KO VCN neurons. We did not observe any difference in AP amplitude or width (determined as full width at half maximum (FWHM)) in Task5 KO neurons as compared to WT controls, independent of the cell’s firing pattern/identity (Figures 2A,B; phasic firing/bushy cells: AP amplitude: WT = 62.5 ± 1.6 mV, *n* = 17 cells/10 mice; KO = 60.4 ± 2.9 mV, *n* = 19 cells/6 mice; *p* = 0.542; unpaired *t*-test; FWHM: WT = 1.18 ± 0.1 ms, *n* = 17 cells/10 mice; KO = 1.47 ± 0.12 ms, *n* = 19 cells/6 mice; *p* = 0.23; unpaired *t*-test; tonic firing/stellate cells: AP amplitude: WT = 100 ± 4.2 mV, *n* = 11 cells/5 mice; KO = 90.2 ± 3.2 mV, *n* = 10 cells/6 mice; *p* = 0.08; unpaired *t*-test; FWHM: WT = 0.92 ± 0.06 ms, *n* = 11 cells/5 mice; KO = 0.73 ± 0.09 ms, *n* = 10 cells/6 mice; *p* = 0.08; unpaired t-test). Moreover, Task5 KO had no impact on the resting membrane potential (RMP) in either type of VCN neurons (Figures 2A,B; phasic firing/bushy cells: RMP: WT = −53.5 ± 1.1 mV, *n* = 17 cells/10 mice; KO = −51.8 ± 0.9 mV, *n* = 19 cells/6 mice; *p* = 0.26; unpaired *t*-test; tonic firing/stellate cells: WT = −56.9 ± 1.5 mV, *n* = 11 cells/5 mice; KO = −55 ± 2.1 mV, *n* = 10 cells/6 mice; *p* = 0.48; unpaired *t*-test).

Next, we investigated the firing properties of both types of VCN neurons and determined their rheobase current, by prolonged current injections (200 ms). We found that phasically firing Task5 KO neurons required significantly more current than WT controls to elicit an AP (Figure 2C, rheobase current: WT = 114 ± 13 pA, *n* = 17 cells/10 mice; KO = 164 ± 11 pA, *n* = 19 cells/6 mice; *p* = 0.006; unpaired *t*-test), while the rheobase current of tonically firing cells was not affected by Task5 KO (Figure 2D, rheobase current: WT = 104 ± 25 pA, *n* = 11 cells/5 mice; KO = 72 ± 21 pA, *n* = 10 cells/6 mice; *p* = 0.35; unpaired *t*-test).

A different effect was seen when we examined the input/output curves of phasically and tonically firing VCN cells. In phasically firing neurons, Task5 KO had no effect on the number of APs fired in response to a given current injection (Figure 2E; recordings from 17 WT and 19 KO neurons from 10 and 6 animals, respectively; two-way ANOVA, genotype *p* = 0.78). However, tonically firing Taks5 KO cells showed slightly increased numbers of APs in response to a given current injection, as compared to WT controls. Thus, tonically firing VCN neurons lacking Task5 had a steeper input/output curve (Figure 2F; recordings from 11 WT and 10 KO neurons from 5 and 6 mice, respectively, two-way ANOVA, genotype *p* < 0.0001). Of note, this effect was relatively small, since we could not detect significant differences at individual current strengths (Figure 2F).

### Effects of Task5 on the cellular properties of MNTB principal neurons

**Figure 3:**
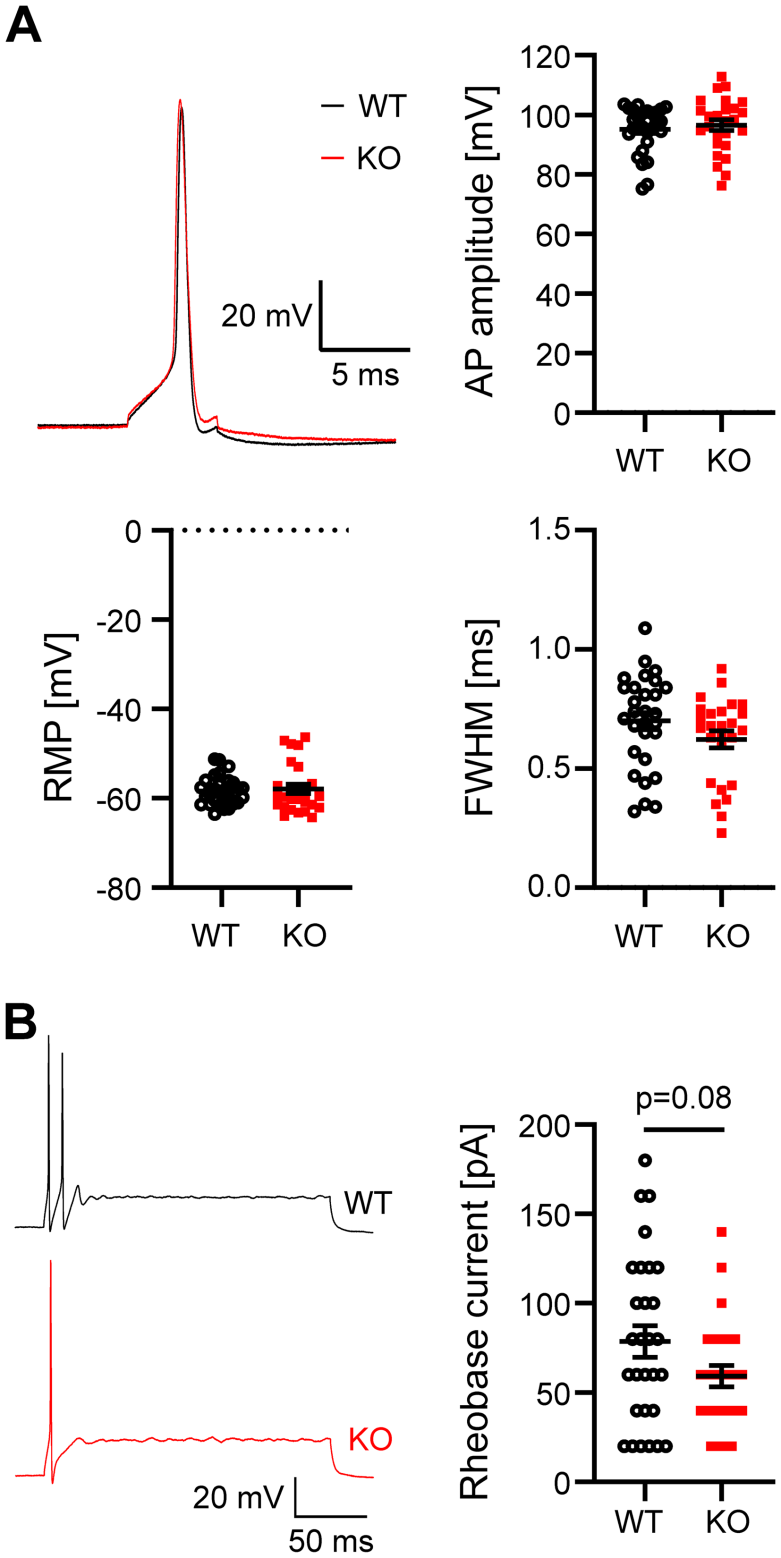
Task5 KO does not affect cellular properties of MNTB principal neurons. **(A)** Representative AP recordings from MNTB principal neurons and quantification of AP amplitude, resting membrane potential (RMP) and AP width [full width at half maximum (FWHM)]. **(B)** Representative recordings of the neuronal firing pattern in response to prolonged depolarizations (left) and quantification of the rheobase current (right). Data is presented as mean ± sem. Statistical testing: unpaired *t*-test.

Task5 is not only expressed in the VCN, but throughout the auditory brainstem, including the SOC (Karschin et al., 2001; Ehmann et al., 2013). Therefore, we next investigated neuronal properties in the MNTB, whose principal cells form a homogeneous population of cells with a phasic firing pattern (Smith et al., 1998; Borst and Soria van Hoeve, 2012). Analogous to the VCN, we first examined the AP amplitude and width as well as the RMP in WT and Task5 KO MNTB principal neurons. Again, we did not observe significant differences in these parameters between genotypes (Figure 3A; AP amplitude: WT = 95.1 ± 1.4 mV, *n* = 29 cells/11 mice; KO = 96.5 ± 1.8 mV, *n* = 26 cells/7 mice; *p* = 0.54; unpaired *t*-test; FWHM: WT = 0.7 ± 0.04 ms, *n* = 29 cells/11 mice; KO = 0.62 ± 0.04 ms, *n* = 26 cells/7 mice; *p* = 0.14; unpaired *t*-test; RMP: WT = −58.3 ± 0.6 mV, *n* = 29 cells/11 mice; KO = −57.9 ± 1.1 mV, *n* = 26 cells/7 mice; *p* = 0.71; unpaired *t*-test). Next, we stimulated MNTB neurons with prolonged current injections and determined the rheobase current. MNTB principal cells responded with a phasic firing pattern to prolonged current injections (Figure 3B). However, in contrast to phasically firing cells in the VCN, MNTB principal neurons from Task5 KO mice required slightly less current to reach the AP threshold than WT ones, although this effect did not reach significance (rheobase current: WT = 79 ± 9 pA, *n* = 29 cells/11 mice; KO = 59 ± 6 pA, *n* = 26 cells/7 mice; *p* = 0.08; unpaired *t*-test) (Figure 3B).

### Functional compensation of Task5 KO

**Figure 4:**
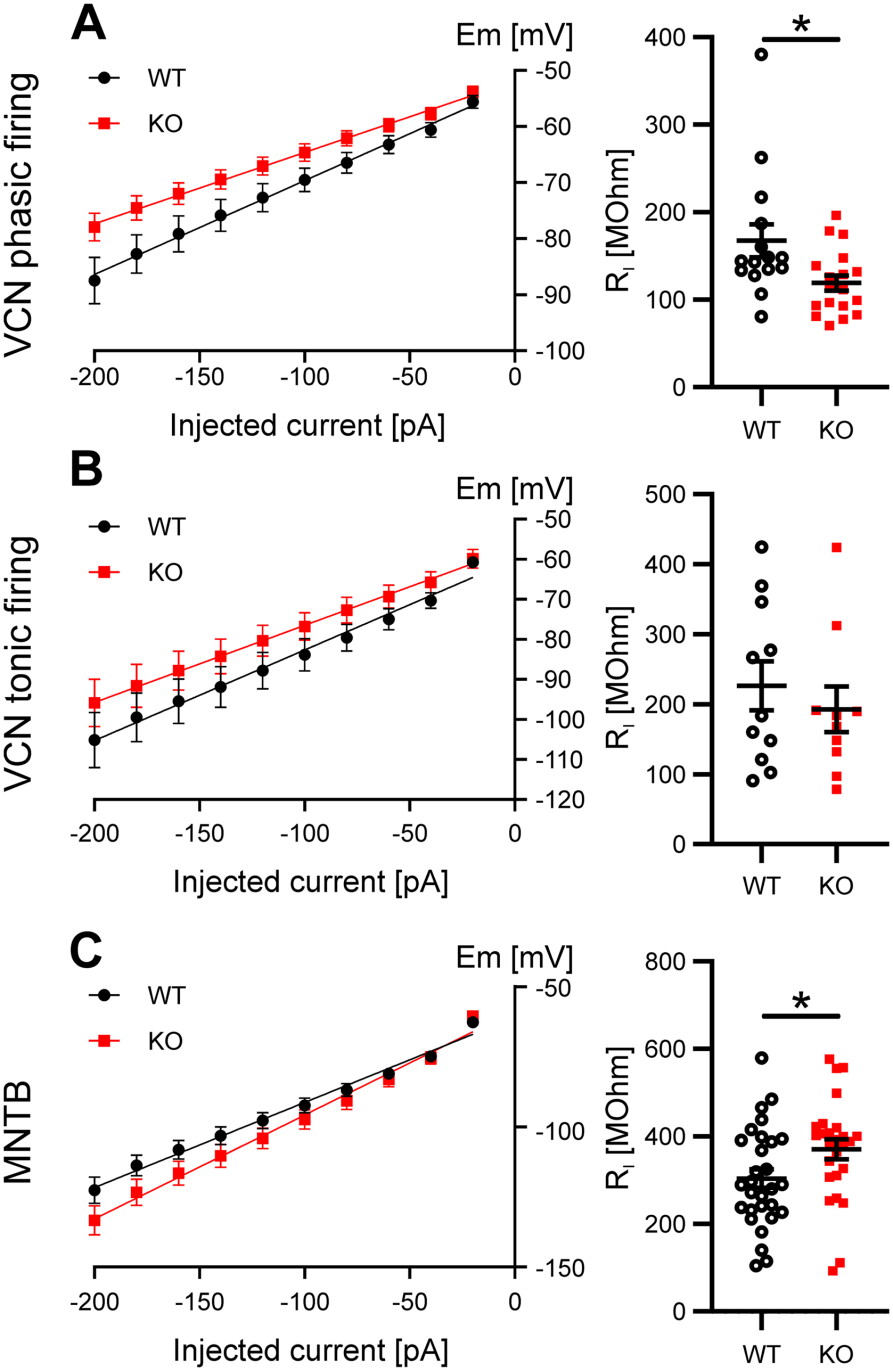
Task5 KO leads to functional compensation by changes in R_I_ in phasically firing VCN neurons and MNTB principal neurons. **(A–C)** V/I plots (left) and quantification of R_I_ (right) obtained from phasically **(A)** and tonically **(B)** firing VCN neurons as well as MNTB principal cells **(C)**. Data is presented as mean ± sem. * = *p* < 0.05, Statistical testing: unpaired *t*-test.

The results presented so far showed only differences in the rheobase current in phasically firing VCN neurons and, to a lesser extent, MNTB principal cells, but no alterations in RMP or AP waveform, which would have been expected for the loss of a K2P subunit. We thus hypothesized that both types of neurons compensate for the loss of Task5 in order to maintain the RMP, AP waveform and firing properties required for their physiological function. To gain further insights into the mechanism(s) by which phasically firing VCN neurons and MNTB principle cells compensate for the absence of Task5, we analysed the input resistance (R_I_) of WT and KO neurons. R_I_ was calculated from I/V curves generated by current injections between-100 and-20 pA (Figure 4). Phasically firing VCN neurons lacking Task5 showed a lower R_I_ as compared to WT controls (WT = 168 ± 19 MΩ, n = 15 cells/9 mice; KO = 119 ± 9 MΩ, *n* = 18 cells/6 mice; *p* = 0.02; unpaired *t*-test; Figure 4A, right), indicating a higher number of open ion channels at rest. In contrast, MNTB principal cells from Task5 KO mice had a slightly higher R_I_ than WT controls (WT = 304 ± 21 MΩ, *n* = 29 cells/11 mice; KO = 371 ± 23 MΩ, *n* = 26 cells/7 mice; *p* = 0.036; unpaired *t*-test; Figure 4C, right), which suggests a lower number of ion channels open at rest. Tonically firing VCN neurons did not show differences in R_I_ between genotypes (WT = 226 ± 35 MΩ, *n* = 11 cells/5 mice; KO = 193 ± 33 MΩ, *n* = 10 cells/6 mice; *p* = 0.49; unpaired *t*-test; Figure 4B, right). Thus, phasically firing neurons seem to counteract the loss of Task5 by regulating their content of ion channels open at rest. However, it is not clear from these experiments which channels are involved in this functional compensation.

**Figure 5:**
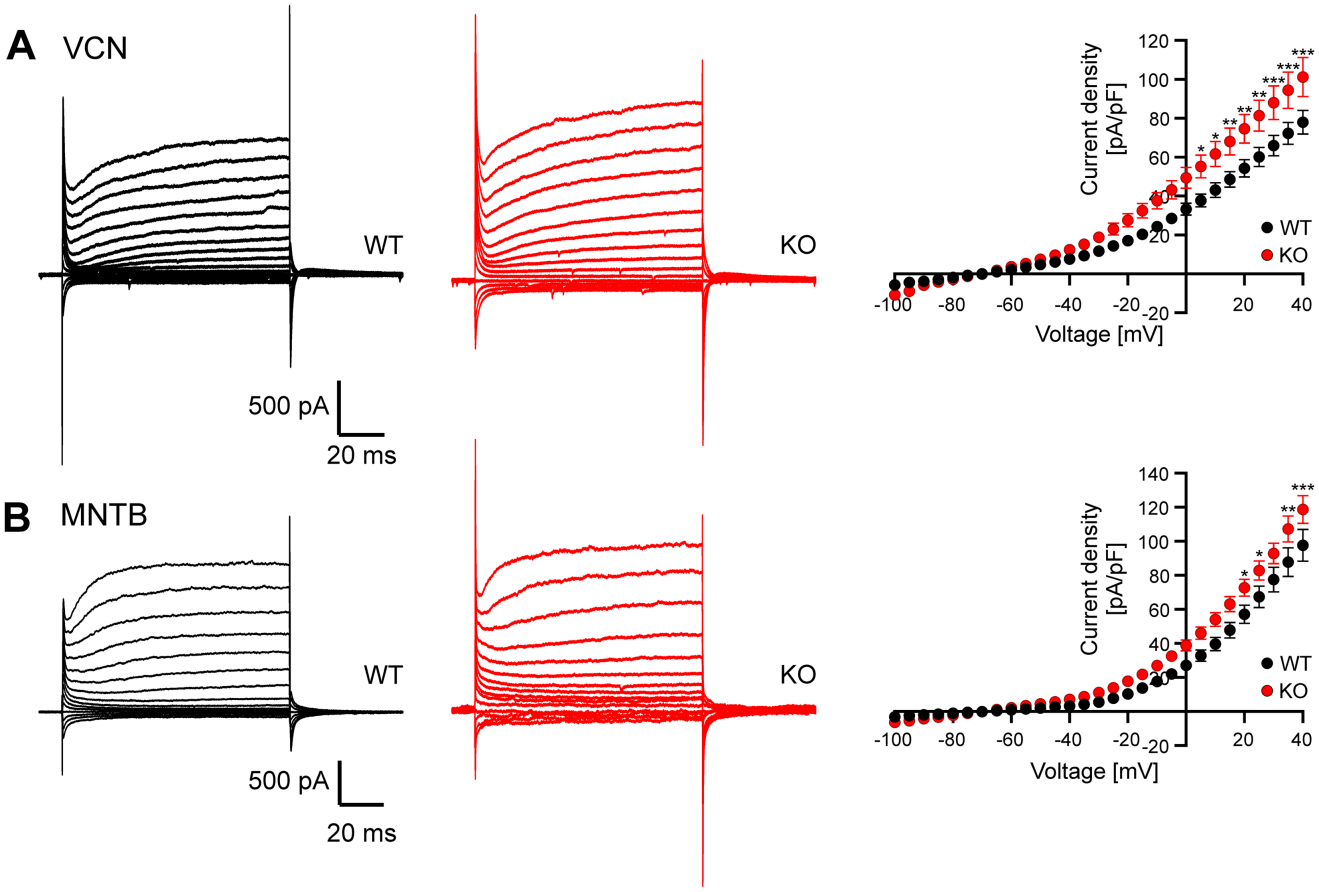
Task5 KO neurons show increased leak conductance. **(A)** Representative current traces in response to voltage steps from −100 mV to +40 mV from a holding potential of −70 mV recorded from WT (left) and Task5 KO (middle) VCN neurons. Right: Current density/voltage plots. **(B)** Representative current traces in response to voltage steps from-100 mV to +40 mV from a holding potential of-70 mV recorded from WT (left) and Task5 KO (middle) MNTB principal neurons. Data is presented as mean ± sem. * = *p* < 0.05, ** = *p* < 0.01, *** = *p* < 0.001, Statistical testing: two-way ANOVA followed by Sidak’s test for multiple comparisons.

To gain further insights into the effects of Task5 KO on the leak conductance of VCN cells and MNTB principal neurons, we next measured leak currents in those cells. Leak conductances were isolated by applying blockers for voltage-gated sodium, potassium and calcium channels as well as HCN channels (see Material and Methods section). Leak currents were measured in response to step depolarizations from −100 mV to +40 mV from a holding voltage of −70 mV. Of note, VCN cells could not be distinguished by there firing pattern anymore. However, we rejected cells showing a rapidly inactivating current component insensitive to TEA-Cl and 4-AP since these cells are most likely stellate cells (Rothman and Manis, 2003; see discussion). Interestingly, we observed larger leak currents in both, VCN neurons and MNTB principal neurons from Task5 KO mice as compared to WT controls, although the effect was less pronounced in MNTB principal cells (Figure 5, VCN: *p* < 0.0001, *F* = 84.73, two-way ANOVA, Sidak’s multiple comparison test, WT: 18 cells from 7 animals; KO: 14 cells from 6 animals; MNTB: p < 0.0001, *F* = 54.41, two-way ANOVA, Sidak’s multiple comparison test, WT: 11 cells from 5 animals; KO: 16 cells from 5 animals). These results are in line with a recent report showing that Task5 is a negative regulator of Task1 and Task3 channels (Rinné et al., 2024).

Our results thus suggest that the observed decrease in R_I_ in phasically firing VCN neurons of Task5 KO mice is due to an increase in leak conductance, probably mediated by the omission of the inhibitory effect of Task5 on other K2P subunits. In MNTB principal cells lacking Task5 the increase in leak conductance seems to be overcompensated. However, since AP properties and RMP are not different between genotypes in either cell type, the neurons seem to compensate for the loss of Task5 and the concomitant increase in leak conductance by different and yet unidentified mechanisms.

### Auditory brainstem responses are altered in Task5 KO mice

**Figure 6:**
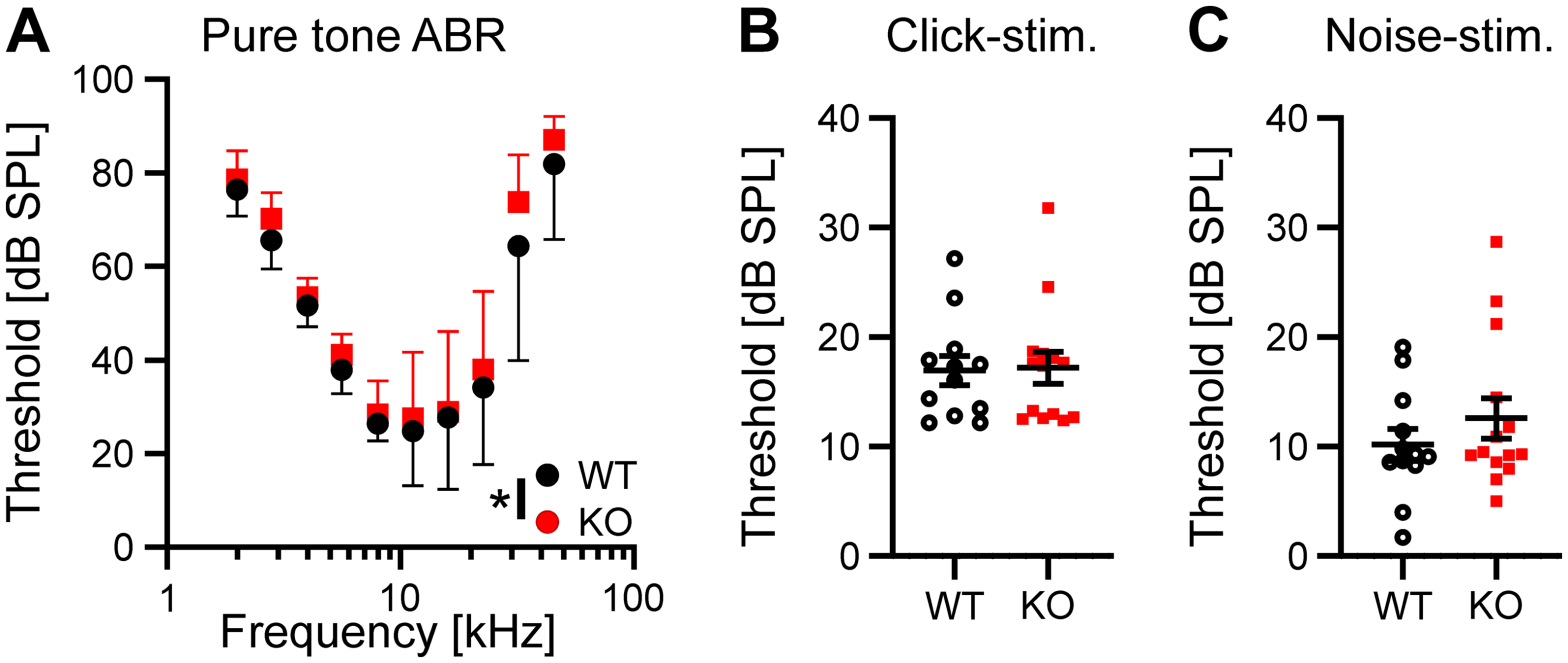
Task5 KO mice show mild alterations in pure tone ABR thresholds. **(A)** Frequency resolved, pure tone thresholds. **(B,C)** Quantification of ABR thresholds in response to click **(B)** and noise stimulation. Data is presented as mean ± sd **(A)** or mean ± sem **(B,C)**. * = *p* < 0.05, Statistical testing: two-way ANOVA followed by Sidak’s test for multiple comparisons **(A)** or unpaired *t*-test **(B,C)**.

**Figure 7:**
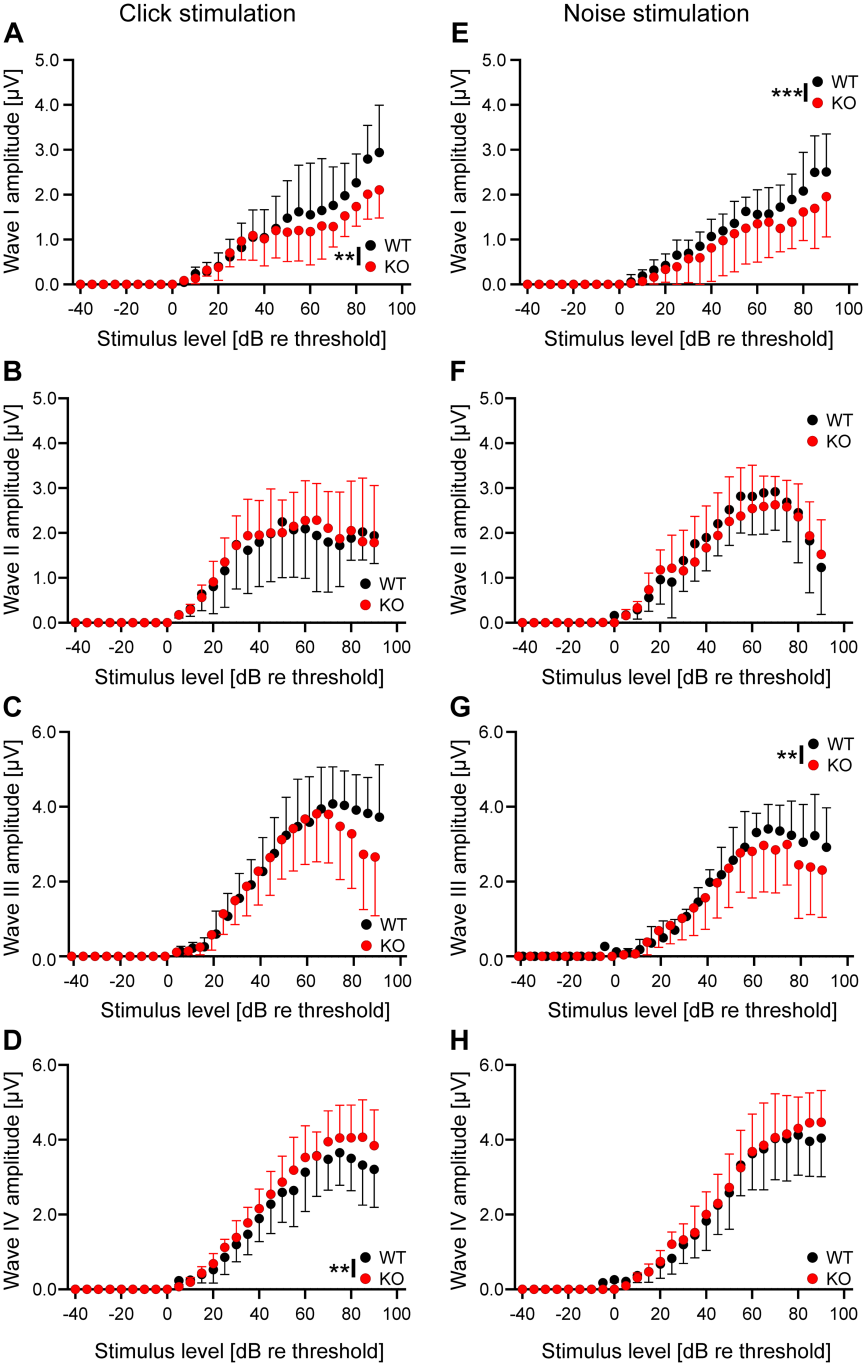
Task5 KO results in changes in ABR amplitudes in response to click and noise stimulation at high SPLs. **(A–H)** Growth functions of wave I **(A,E)**, wave II **(B,F)**, wave II **(C,G)** and wave IV **(D,H)** amplitudes in response to click **(A–D)** and noise **(E–H)** stimulation, relative to threshold. Data is presented as mean ± sd. ** = *p* < 0.01, *** = *p* < 0.001, Statistical testing: two-way ANOVA followed by Sidak’s test for multiple comparisons.

The results described above suggest that neurons in the auditory brainstem are only mildly affected by the loss of Task5, possibly due to functional compensation. We asked whether the small changes observed in individual Task5 KO neurons could sum up on the systemic level and affect auditory processing *in vivo*. Therefore, we measured auditory brainstem responses (ABRs) in 6 to 8 weeks-old Task5 KO mice and WT littermate controls, at which age ABRs have become stable. ABR measurements showed normal thresholds for Task5 KO mice in response to click as well as noise stimuli (Figures 6B,C; click stimuli: WT = 16.97 dB SPL ± 1.33 dB, *n* = 12 ears from 6 mice; KO = 17.21 dB SPL ± 1.47 dB, *n* = 14 ears from 7 mice; *p* = 0.91; unpaired *t*-test; noise stimuli: WT = 10.18 dB SPL ± 1.45 dB *n* = 12 ears from 6 mice; KO = 12.59 dB SPL ± 1.85 dB, *n* = 14 ears from 7 mice; *p* = 0.33; unpaired *t*-test; SPL = sound pressure level). However, frequency resolved ABR measurements in response to pure tone stimulation revealed mild changes in hearing thresholds in KO mice compared to WT controls. The biggest apparent differences were seen in the high frequency domain (32 kHz). Of note, we did not observe a significant difference for individual frequencies in post hoc tests (Figure 6A; *p* = 0.014, *F* = 6.18, two-way ANOVA, Sidak’s multiple comparison test, WT: *n* = 12 ears from 6 mice, KO: *n* = 14 ears from 7 mice; Table 1). Since the individual waves of ABRs can be attributed to synchronous neuronal activity along the auditory pathway (wave I = auditory nerve fibers, wave II = CN, wave III = SOC, wave IV = combined activity of the lateral lemniscus and the inferior colliculus; Melcher and Kiang, 1996; Rüttiger et al., 2017), we performed a detailed analysis of the click-and noise-induced ABR waves. For both types of stimuli, we observed reduced amplitudes of wave I at high SPLs in Task5 KO mice compared to WT controls, while the amplitude of wave II was unaltered (click stimuli: Figures 7A,B; ABR wave I: *p* = 0.0038, *F* = 8.48; ABR wave II: *p* = 0.68, *F* = 0.171; two-way ANOVA, Sidak’s multiple comparison test, WT: *n* = 12 ears from 6 mice, KO: *n* = 14 ears from 7 mice; Table 1; noise stimuli: Figures 7E,F; ABR wave I: *p* = 0.0002, *F* = 17.46; ABR wave II: *p* = 0.42, *F* = 0.666; two-way ANOVA, Sidak’s multiple comparison test, WT: *n* = 12 ears from 6 mice, KO: *n* = 14 ears from 7 mice; Table 1). The amplitude of wave III was significantly reduced in Task5 KO mice upon application of noise stimuli at high SPLs (Figure 7G; ABR wave III: *p* = 0.0055, *F* = 7.827; two-way ANOVA, Sidak’s multiple comparison test, WT: *n* = 12 ears from 6 mice, KO: *n* = 14 ears from 7 mice; Table 1). Click stimuli resulted in lower wave III amplitudes in Task5 KO mice, too, although this reduction did not reach significance (Figure 7C; ABR wave III: *p* = 0.22, *F* = 1.497; two-way ANOVA, Sidak’s multiple comparison test, WT: *n* = 12 ears from 6 mice, KO: *n* = 14 ears from 7 mice; Table 1). In contrast to the amplitude reductions in waves I and III, we observed increased wave IV amplitudes in Task5 KO mice, although this effect only reached significance for click stimuli (click stimuli: Figure 7D, ABR wave IV: *p* = 0.0024, *F* = 9.382; two-way ANOVA, Sidak’s multiple comparison test; WT: *n* = 12 ears from 6 mice, KO: *n* = 14 ears from 7 mice; Table 1; noise stimuli: Figure 7H, ABR wave IV: *p* = 0.17, *F* = 1.903; two-way ANOVA, Sidak’s multiple comparison test; WT: *n* = 12 ears from 6 mice, KO: *n* = 14 ears from 7 mice; Table 1). Together, the changes in ABRs in Task5 KO mice show that the functional compensation observed on the cellular level is insufficient to completely restore proper auditory processing at the systemic level. In particular the processing of loud sounds is hampered in the absence of Task5.

**Table 1:** Statistics of ABR analysis: results for two-way ANOVAs and Sidak’s post hoc tests.

Parameter	Test	df	Effect	*F*	*p*	Sidak’s multiple comparisons test
f-ABR	WT vs. KO	1, 238	Frequency Genotype Interaction	94.69 6.181 0.2792	< 0.0001 0.0136 0.9799	n.s.
Click ABR						
Wave I	WT vs. KO	1, 346	Sound intensity Genotype Interaction	19.96 8.476 1.054	< 0.0001 0.0038 0.398	90 dB: 0.05
Wave II	WT vs. KO	1, 349	Sound intensity Genotype Interaction	7.587 0.1714 0.3060	< 0.0001 0.6791 0.9969	n.s.
Wave III	WT vs. KO	1, 323	Sound intensity Genotype Interaction	31.51 1.497 0.5305	< 0.0001 0.2221 0.9371	n.s.
Wave IV	WT vs. KO	1, 322	Sound intensity Genotype Interaction	62.44 9.382 0.3067	< 0.0001 0.0024 0.9968	n.s.
Noise ABR						
Wave I	WT vs. KO	1, 259	Sound intensity Genotype Interaction	17.46 14.66 0.4136	< 0.0001 0.0002 0.9817	90 dB: 0.06
Wave II	WT vs. KO	1, 297	Sound intensity Genotype Interaction	17.72 0.6661 0.3477	< 0.0001 0.4151 0.9964	n.s.
Wave III	WT vs. KO	1, 325	Sound intensity Genotype Interaction	29.94 7.827 0.5184	< 0.0001 0.0055 0.9435	n.s.
Wave IV	WT vs. KO	1, 322	sound intensity Genotype Interaction	68.33 1.903 0.2115	< 0.0001 0.1686 0.9997	n.s.

## Discussion

In the present study, we examined the function of the K2P subunit Task5 in the auditory brainstem. Using a constitutive KO mouse line, we show that Task5 contributes to auditory processing on the cellular as well as the systemic level. Although phasically firing neurons seemed to functionally compensate for the loss of Task5, we describe changes in rheobase current, input resistance and leak conductance in both MNTB principal cells and phasically firing VCN neurons, most likely bushy cells. Moreover, the small changes apparent on the cellular level still led to alterations in ABR wave amplitudes on the systemic level.

### Developmental expression of Task5 and its relation to hearing

Task5 expression is tightly regulated during development. In juvenile neurons, before the onset of hearing, it is expressed at low levels, both in GBCs and in the SOC (Körber et al., 2014; Ehmann et al., 2013, respectively). Gene expression studies have shown that Task5 is upregulated between P4 and P16 in the SOC (Ehmann et al., 2013) and between P8 and P21 in GBCs (Körber et al., 2014). The combined data from both studies thus suggests a quite abrupt upregulation around the onset of hearing at ~P10-12. Task5 expression is not regulated throughout the prehearing stages of development (SOC: between P0 and P4 (Ehmann et al., 2013); GBCs: between P3 and P8 (Körber et al., 2014)). Moreover, the expression level of Task5 is constant after P16 (SOC, Ehmann et al., 2013, their Figure 6) suggesting the establishment of the mature expression level at or shortly after the onset of hearing. This in turn is inline with auditory brainstem neurons being largely mature, with respect to their cellular properties, shortly after the onset of hearing (e.g., Borst and Soria van Hoeve, 2012; Kandler et al., 2009). Changes in the auditory brainstem circuit that occur after the onset of hearing, and contribute to the stabilization of ABRs, are largely related to the refinement of synaptic connections, e.g., by synaptic pruning, especially in inhibitory connections (Kandler et al., 2009). Thus, we conclude that the cellular recordings presented here reflect a rather mature neuronal state. Despite the tight regulation of Task5 expression, studying its role in the development of the auditory brainstem circuitry, both pre-and post-hearing, will provide an interesting topic for future studies.

In addition to the developmental coincidence between the upregulation of Task5 and the onset of hearing, Task5 expression shows another striking correlation to the perception of airborne sounds: it is rapidly downregulated in the cochlear nucleus and the inferior colliculus of deafened animals (Holt et al., 2006; Cui et al., 2007; Dong et al., 2009). This downregulation occurs within 3 days after cochlear ablation and does not recover for at least 3 months (Holt et al., 2006; Cui et al., 2007), suggesting that Task5 expression depends on the ability to hear. Interestingly, unilaterally deafened animals show downregulation of Task5 only in the ipsilateral cochlear nucleus, while the inferior colliculus is affected bilaterally (Dong et al., 2009). Deaf animals lack the high neuronal firing rates associated with the perception of sound (e.g., Kopp-Scheinpflug et al., 2008). Therefore, it is tempting to speculate that Task5 expression is both, induced by such activity patterns and necessary to correctly process them. We would thus predict that neonatally deafened animals, which never experience such activity patterns, will not express Task5 at all. However, further studies are needed to test this.

### The role of Task5 in auditory brainstem neurons

The KO of a K2P subunit is expected to result in a shift in the RMP, as K2Ps provide the main K^+^-conductance at rest. However, we did not observe such a shift in any of the examined neuronal populations. This could be due to two reasons: either Task5 is indeed a silent channel subunit, or the neurons are functionally compensating for the lack of Task5. We favor the second possibility since we observed changes in the rheobase current, R_I_ and the leak conductance in phasically firing neuron types. These cells, the MNTB principal cells and the phasically firing VCN cells (bushy cells; Rothman and Manis, 2003; Manis et al., 2019) are involved in the high frequency, high-fidelity signal transmission required for sound source localization (Grothe et al., 2010). Interestingly, the establishment of the mature firing pattern of bushy cells as well as MNTB principal cells coincides with the upregulation of Task5 around the onset of hearing (Sonntag et al., 2011; Crins et al., 2011; Ehmann et al., 2013; Körber et al., 2014). The observed shifts in the rheobase current in bushy cells and MNTB neurons of KO mice were accompanied by changes in R_I_ in those cells, indicating that Task5 KO neurons had adjusted the amount of ion channels open at RMP to keep a level of excitability suitable for their physiological task, i.e., the fast and temporally precise transmission of timed signals. Interestingly, we observed an increase in leak conductance in both types of neurons although the R_I_ was regulated in different directions. This increase in leak conductance is in line with a very recent study showing that Task5 forms heteromeric channels with both Task1 and Task3, which have lower surface expression and single channel conductance compared to Task1 and Task3 homomeric channels (Rinné et al., 2024). Of note, a transient increase in potassium leak conductance has also been shown to underly depolarizing after-potentials in neurons of the supraoptic nucleus (Li and Hatton, 1997). In our study, the increase in leak conductance upon Task5 KO could thus explain the decrease in R_I_ in phasically firing VCN neurons. Of note, we could not classify VCN cells based on their firing pattern due to the use of TTX, when recording leak conductances. However, tonically firing VCN neurons were unaffected by Task5 KO in all parameters examined, suggesting that we are rather underestimating the effect of Task5 KO on phasically firing VCN neurons. In contrast to VCN neurons, MNTB principal cells show an increase in R_I_, which probably refelcts an overcompensation for the increase in leak conductance. Nevertheless, both compensatory mechanisms allow the KO neurons to fire WT-like APs and establish a RMP that does not differ from the WT condition. The exact details of functional compensation in VCN cells as well as in MNTB principal cells provide interesting topics for future studies. Such studies would also benefit from a possibility to acutely inactivate Task5, e.g., by a specific inhibitor, which, to the best of our knowledge, is currently not available, probably due to the classification of Task5 as “non-functional.”

Moreover, potassium Trek1 and Traak, two other K2Ps, have recently been shown to mediate AP repolarization in nodes of Ranvier in peripheral nerves (Kanda et al., 2019) suggesting that AP repolarization could be a part of K2P channel function. Although, we did not observe Task5-dependent changes in the AP properties in any of the examined neuron types, we cannot formally exclude a contribution of Task5 to AP repolarization that is masked by functional compensation.

### The systemic role of Task5 in auditory signal processing

In addition to the effects of Task5 KO on the cellular level, we also investigated Task5 function on the systemic level. Therefore, we analysed ABRs of Task5 KO and WT mice in response to noise and click stimuli at 6–8 weeks of age. Although we recognize the age discrepancy between ABR recordings and cellular measurements, both types of experiments examine the largely mature state of either the single neuron’s firing properties or the auditory circuit (see above). Furthermore, ABRs are instable immediately after the onset of hearing due to ongoing synaptic refinement and changes in inhibition, which prevent earlier ABR measurements. Task5 KO animals have almost normal hearing thresholds. Only frequency resolved pure tone ABRs show a small increase in hearing threshold, mostly at high frequencies (Figure 5A). However, we found robust changes in the amplitudes of ABR waves I, III and IV, which occurred specifically at high SPLs (Figure 6), independent of the stimulus type. Click and noise stimuli both have a broad frequency spectrum, but with complementary contributions of high and low frequency components. While noise stimuli comprise more of the high frequencies, click stimuli have a bigger low-frequency component. Although the direction of changes in the ABR wave amplitudes in Task5 KO mice was independent of the stimulus type applied, some effects only reached significance for one of the stimuli (Figure 7). However, significant alterations were detected for both stimulus types, suggesting different interindividual variability in coding strategy as the source of the deviations between stimuli rather than a general problem in the processing of either of the stimuli.

The ABR amplitude represents the synchronous first AP of the cells in a distinct auditory brain region in response to a sound stimulus (Melcher and Kiang, 1996; Rüttiger et al., 2017). Hence, the observed reduction in amplitude for waves I and III suggests a reduced AP synchronicity in the auditory nerve and the SOC of Task5 KO mice. Therefore, Task5 may play a role in the precise timing of AP firing in these two brain regions. Interestingly, wave II, which represents AP firing in the cochlear nuclei, was unaffected by Task5 KO, independent of the stimulus presented. However, the cochlea nuclei are diverse brain regions with a multitude of cell types (e.g., Manis et al., 2019), not all of which may express Task5 physiologically. Here, the normal firing of neuronal cell types that do not express Task5, and are thus unaffected by its KO, may mask potential effects of desynchronized bushy cell firing, leading to apparently normal wave II amplitudes. Finally, we observed an increase in the amplitude of wave IV in Task5 KO mice (Figures 7D,H). This was rather unexpected since it indicates an increase in the synchronicity of AP firing in the lateral lemniscus and/or the inferior colliculus, despite the less precise AP timing in the auditory nerve and the SOC. However, such an increase in wave IV amplitude and a concomitantly smaller wave I amplitude have recently been attributed to central, homeostatic adjustments after mildly traumatic sound exposure (Matt et al., 2018). The increase in wave IV may thus represent an additional, systemic aspect of functional compensation for the loss of Task5.

## Conclusion

Task5 has long been regarded as non-functional (Karschin et al., 2001; Ashmole et al., 2001; Kim and Gnatenco, 2001), although a recent study has shown that Task5 forms heteromeric channels with Task1 and Task3 that possess reduced gating and surface expression compared to homomeric Task1 and Task3 channels (Rinné et al., 2024). The data presented here, show that Task5 is involved in the regulation of cellular excitability and the processing of sounds in the auditory brainstem circuitry. Task5 KO resulted in changes in the rheobase current in phasically firing neurons of the VCN (bushy cells) and the MNTB. These changes in rheobase current were accompanied by changes in R_I_. This represents a functional compensation that differed mechanistically between MNTB principal cells and phasically firing VCN neurons, since both cell types showed an increase in leak conductance in Task5 KO mice. However, the compensatory adjustments on the cellular level were apparently insufficient to completely maintain auditory signal processing on the systemic level. This was particularly evident for the processing of loud sounds, which requires both, high frequency and high-fidelity firing. Although our results obtained in Task5 KO mice are in contrast to previous attempts in heterologous expression systems (Karschin et al., 2001; Ashmole et al., 2001; Kim and Gnatenco, 2001), they are in accordance with a recent study showing that Task5 is required to form heteromeric channels with either Task1 or Task3 to reach the cell surface where its incorporation into the K2P channel results in smaller potassium leak currents due to altered channel gating (Rinné et al., 2024). It remains unclear, whether additional interaction partner(s) are involved in these heteromeric channel complexes and thus further studies on the composition of Task5-containing K2P channels in their physiological environment are needed. In summary, our results provide a first insight into the function of this enigmatic ion channel subunit in the auditory brainstem and relate its highly specific expression pattern to sound processing in the auditory brainstem circuitry.

## Data Availability

The original contributions presented in the study are included in the article/supplementary material, further inquiries can be directed to the corresponding author.
